# Predictive Role of IL-2R and IL-10 in the Anti-inflammatory Response and Antiplatelet Therapy of Kawasaki Disease: A Retrospective Study

**DOI:** 10.1155/2022/4917550

**Published:** 2022-02-03

**Authors:** Chun Zhang, Lun Chen, Sun Chen, Yan Bian, Jia Shen, Peng Zhang, JiaNi Song

**Affiliations:** ^1^Department of Pharmacy, Xinhua Hospital Affiliated to Shanghai Jiao Tong University School of Medicine, Shanghai 200092, China; ^2^Department of Pediatric Cardiology, Xinhua Hospital Affiliated to Shanghai Jiao Tong University School of Medicine, Shanghai 200092, China; ^3^Department of Laboratory Medicine, Xinhua Hospital Affiliated to Shanghai Jiao Tong University School of Medicine, Shanghai 200092, China; ^4^Department of Pharmacy, Shanghai University of Medicine &Health Sciences, Shanghai 201318, China

## Abstract

To date, Kawasaki disease (KD) has only been able to be diagnosed and evaluated using clinical characteristics. Additionally, the therapeutic effect and cardiovascular complications could not be verified until its occurrence. The present retrospective study analyzed the dynamic alterations of inflammatory cytokines, platelet (PLT) count, and subgroups of lymphocytes, such as cluster of differentiation (CD) 8^+^ T cells and CD19^+^ B cells, under different conditions in 64 children with KD. The percentage distribution of lymphocyte subgroups and the altered neutrophil lymphocyte ratio demonstrated that the inflammatory response was dominated by the B cell-mediated humoral immune response before intravenous immunoglobulin (IVIG) treatment, but mainly by T cells via cellular cytotoxic effects after IVIG treatment. Among the different types of inflammatory cytokines, the results of the present study revealed that the altered levels of interleukin-2 receptor (IL-2R) and interleukin-10 (IL-10) were closely associated with the percentage of CD8^+^ T cells and CD19^+^ B cells. Additionally, the two cytokines exhibited more sensitive fluctuations based on the status of the children with KD in various circumstances compared with other indexes, such as the percentages of CD8^+^ T cells and CD19^+^ B cells or the PLT count. These results suggested that children with KD who are ≥4 years old may benefit from IVIG but will not benefit from decreased platelet activation or suffer less cardiovascular complications. Additionally, starting clopidogrel usage earlier as an antiplatelet strategy should be considered based on the observed continuous rise in the PLT count in children with KD receiving IVIG. In conclusion, dynamically monitoring the levels of IL-2R and IL-10 has the potential to provide indications of the intensity and development of the inflammatory response in children with KD and may contribute to the early prediction and adjustment of pathological and pharmacological effects of therapy.

## 1. Introduction

Kawasaki disease (KD) was formally reported over 50 years ago. With the development of a therapeutic regimen, particularly timely high-dose intravenous immunoglobulin (IVIG) usage in the acute phase, the mortality of KD has significantly declined. However, 7.9% of patients still experience cardiovascular complications, including coronary dilation, valve lesions, coronary aneurysms, coronary stenosis, and acute myocardial infarction [1, 2]. KD predominantly affects children, and disease-related cardiovascular complications are the most common causes of acquired heart disease in children in developed countries as reported [1, 3]. In recent years, with the accumulative experience and consciousness of KD, higher occurrences of coronary artery lesions in KD were reported than that of before. In China, cardiovascular complications of KD have gradually become the main cause of acquired heart disease. Nowadays, in both developed countries and developing countries including the United States, Japan, and China, cardiovascular complications from KD have been considered consistently to be the main cause of acquired heart disease [1, 3–6].

Clinically, KD is an acute, self-limiting febrile illness. Essentially, KD is an inflammatory response within the middle and small blood vessels throughout the entire body. Previous studies have reported the importance of several genetic factors, geographic heterogeneity, and immunity-associated inflammation in the pathogenesis of KD, including the role of B cells and interleukin (IL)-1*β* [7, 8]. These findings attempted to help elucidate the inflammatory mechanism in children with KD. However, at present, there are no common laboratory parameters that can clearly delineate the development of the inflammatory status in patients with KD, including those who are being treated. This is one of the main problems in KD, which not only impedes the evaluation of the disease but also delays the adjustment of subsequent therapies.

An increased number of studies have begun to focus on the status of inflammatory cytokines in KD, and accumulating evidence has revealed the critical role of inflammatory cytokines in different aspects of KD. The dynamic levels of cytokines during the course of KD disease progression and the underlying mechanism may provide children with KD with novel opportunities, especially in terms of the possible prediction of disease prognosis and novel treatment options. According to previous reports, IL-6 is a potential biomarker for predicting the resistance of IVIG [9, 10]. One of the effects of immunomodulation with IVIG is the inhibition of IL-10 secretion, which is the result of depressed B cell proliferation and function [11] and consistent with the results of our previous study [12] and the current study findings. In addition, the course of KD may benefit from a strategy for dealing with certain proinflammatory cytokines including deleting their receptors genetically or usage of their blockade, such as tumor necrosis factor (TNF), by aiming at which the protective effects on KD vasculitis have been verified in animal models [13]. The promotion of IL-1*β*-induced KD vasculitis in mice led to the initiation of clinical trials assessing the effect of anakinra for blocking IL-1*β* as a secondary therapy option to treat children with IVIG-resistant KD [14–16]. Suppression of IL-2 and thus leading to the inhibition of T cell activation contribute to a decreased incidence of coronary artery abnormalities [17]. However, not every novel treatment can be applied to all patients with KD. Appropriate selection of individual patients with typical characteristics of altered laboratory parameters is a more feasible strategy. Therefore, the present study investigated the inflammatory cytokines, the associated distribution of lymphocyte subgroups, and platelet (PLT) count in children with KD under different conditions. The present study was aimed at describing the characteristic profile of cytokines in children with KD under different conditions to elucidate the possibility of the predictive and evaluative role of certain cytokines in the treatment of KD.

## 2. Materials and Methods

### 2.1. Aim, Design, and Setting of the Study

The present study was retrospective, and the medical record data of all 64 children with KD were collected with the permission of the Ethics Committee of Xinhua Hospital Affiliated to Shanghai Jiao Tong University School of Medicine (Shanghai, China). The included medical data of children with KD were obtained from the past medical history and the laboratory database. The present study was approved by the Ethics Committee of the Xinhua Hospital (approval no. XHEC-D-2021-007). The data included in this research are available from Xinhua Hospital but are not publicly available because of the restrictions in acquiring the data. If there is a reasonable request, information could be obtained from the authors with the permission of Xinhua Hospital.

The normal ranges of healthy children were provided by the Department of Laboratory Medicine at Xinhua Hospital.

### 2.2. Patients

A total of 64 children diagnosed with KD between January 2019 and March 2021 at Xinhua Hospital Affiliated to Shanghai Jiao Tong University School of Medicine (Shanghai, China) were enrolled in this retrospective study. The diagnosis, laboratory detections, and therapeutic treatment were performed according to the Guidelines from the Scientific Statement for Health Professionals from the American Heart Association [3]. The clinical criteria of diagnosis were as follows: the presence of ≥5 days of fever and the presence of ≥4 of 5 principal clinical features of KD. Considering the typical clinical features or clinicians' abundant experience in treating KD, sometimes the children could be diagnosed earlier, even if the presence of fever was only 3-4 days. Patients were excluded from the study based on the following criteria: (1) incomplete clinical medical data; (2) presence of serious cardiovascular, hepatic, or renal diseases and primary disease associated with tumors, hematological diseases, congenital malformations, genetic metabolic diseases, primary myocarditis, or other primary diseases of major organs; and (3) relapse that required retreatment.

According to the different requirements of the study, the children with KD were grouped as follows: (1) younger children with KD who were <4 years old and older children with KD aged ≥4 years; (2) children with KD treated with and without clopidogrel; (3) before IVIG (laboratory parameters of the blood samples obtained and detected at 24 h before the performance of IVIG) and after IVIG (laboratory parameters of the blood samples obtained and detected at 72 h after IVIG); and (4) complete KD and incomplete KD (the patients' medical records were reviewed and evaluated according to the statement for health professionals from the American Heart Association about the diagnosis, treatment, and long-term management of KD [3]).

### 2.3. Procedures and Samples

On the basis of the guideline “Diagnosis, Treatment, and Long-Term Management of Kawasaki Disease: A Scientific Statement for Health Professionals from the American Heart Association” [3], when children were diagnosed with KD, IVIG was applied within 24 h (single dose of 2 g/kg). The blood samples of children with KD were collected 24 h before IVIG treatment for the detection of lymphocyte subgroup counts using a flow cytometer according to the requirement of clinical performance (BD CANTO Plus; BD Biosciences). Cytokines were tested before and after IVIG using a solid-phase enzyme-labeled chemiluminescent immunometric assay (Siemens AG).

About the usage of corticosteroids, in line with the statement recommended [3], children coming to Xinhua Hospital once diagnosed with KD would be evaluated with echocardiography before and after IVIG; when the patients were identified as coronary artery abnormalities, the adjunctive therapy of corticosteroids will be administrated to patients.

About the application of IVIG retreatment, in accordance with the statement from AHA [3], the child with KD who had persistent fever or recrudescent fever for at least 36 hours would receive IVIG retreatment after being infused with the first dose of IVIG.

For the usage of clopidogrel, based on the patients' clinical features and with the permission of both Ethics Committee of Xinhua Hospital and Pharmaceutical Affairs Committee of Xinhua Hospital according to the guide published during 2017 to 2021 [1, 3, 4], children with KD in two cases could be treated with combinative regimen of clopidogrel, IVIG, and aspirin. One is that after IVIG, the patient's PLT count rises persistently to a higher level than that of 600 × 10^9^/L, the other is the dilation of coronary artery luminal is found in the patient by echocardiography after IVIG.

After IVIG, there were several times of tests to monitor the dynamic change of PLT count with consideration of the patients' clinical features. When the count of PLT rose higher than 600 × 10^9^/l, clopidogrel would be administered to the patients. The data of the PLT count just prior to the usage of clopidogrel was chosen in the present study.

### 2.4. Statistical Analysis

All statistical analyses were performed using SPSS 22.0 (IBM Corp.), GraphPad Prism 6.0 (GraphPad Software, Inc.), and Excel 2010 (Microsoft Corporation) softwares. Continuous variables are presented as the mean ± standard deviation, percentiles as median and range (P_25_-P_75_), and other data as percentage (%) and absolute number (*n*). For some variables, a logarithmic transformation was performed to make the data conform to a normal distribution. Groups were compared using the Student *t*-test, nonparametric Mann-Whitney test, Wilcoxon test, Fisher's exact test, or Welch's *t*-test. The correlation between two indexes, such as cytokines and/or subgroups of lymphocytes, was analyzed using Spearman's coefficient. The threshold for statistical significance was set to *P* < 0.05 (^∗^*P* < 0.05; ^∗∗^*P* < 0.01; ^∗∗∗^*P* < 0.001; ^∗∗∗∗^*P* < 0.0001).

## 3. Results

### 3.1. Characteristics of Children with KD

Table 1 shows the characteristics of the 64 children with KD included in the present study. There were 41 male patients (64.1%) and 23 female patients (35.9%) included in the study, and no difference in the sex distribution was found between these patients and those that were newly reported [1], in which study, there was a total of 31595 patients with KD consisting of 18060 male and 13535 female. The neutrophil lymphocyte ratio (NLR) of all patients after IVIG treatment fell within the normal range of values, which was <5.0 under physiological conditions in the study [18]. Notably, prior to IVIG treatment, the percentage of cluster of differentiation (CD)19^+^ B cells in children with KD was markedly higher than the normal value, while that of CD8^+^ T cells was lower compared with the physiological value established by our hospital based on the data of healthy children (*n* ≥ 200). The levels of IL-10 before IVIG and IL-2 receptor (IL-2R) both before and after IVIG were higher than the normal values.

According to our previous study, an improved effect of IVIG on the immune response and immune equilibrium of children with KD was observed in those who were ≥4 years [12]. Therefore, in the present study, children with KD were first divided into two groups: children who were <4 years old and children aged ≥4 years (Table 2). In addition to the obvious differences in age and body weight between the two groups, the percentage of CD19^+^ B cells in children with KD who were <4 years old was markedly higher than that in children with KD aged ≥4 years. Furthermore, the percentage of CD8^+^ T cells of older children with KD was higher than that of the younger patients. When comparing the two groups, the extent of the decrease of both IL-2R and IL-10 in the younger children with KD was markedly higher than that in the older children with KD.

The clinical features of both complete KD cases and incomplete ones are listed in Supplementary Table S1. In the 64 cases involved in the present study, between complete KD and incomplete KD, there were no obvious differences in the items including gender, age, duration of persistent fever till the diagnosis of KD, duration of fever till receiving the first dose of IVIG, and the ratio of coronary artery abnormalities.

The numbers of patients are listed in Supplementary Table S2, who occurred the main symptoms at diagnosis among the children with complete and incomplete KD. By comparison, in children with complete KD, the symptoms of erythematous rash in the trunk and cervical lymphadenopathy were more predominant, while extremity changes appeared less frequently than those in children with incomplete KD.

For patients who received IVIG retreatment or adjunctive therapy of corticosteroids, detailed information about the therapeutic regimen and the relevant timing is listed in Supplementary Table S3-5.

In both children with complete KD and incomplete KD, IVIG was administered to patients on the 6th day (4.63-7) averagely after the fever onset. Supplementary Table S3 showed the mean time of persistent fever before the patients receiving the second or third dose of IVIG. Supplementary Table S4 showed the individual characteristics of patients who received IVIG retreatment, such as the duration of fever till diagnosis or the lasting time of persistent fever before the retreatment of IVIG.

Supplementary Table S5 lists the individual characteristics of patients receiving adjunctive therapy of corticosteroids. According to the guideline published by AHA in 2017 [3], the addition of corticosteroid therapy with the standard dose of IVIG and low dose of aspirin in the primary therapy of KD lowers the prevalence of coronary artery abnormalities. In the present study, the adjunctive therapy of corticosteroids was given to four patients when they were identified as coronary artery abnormalities by echocardiography.

### 3.2. Effect of IVIG on the Inflammatory Response of Children with KD in Different Age Groups

**Figure 1:**
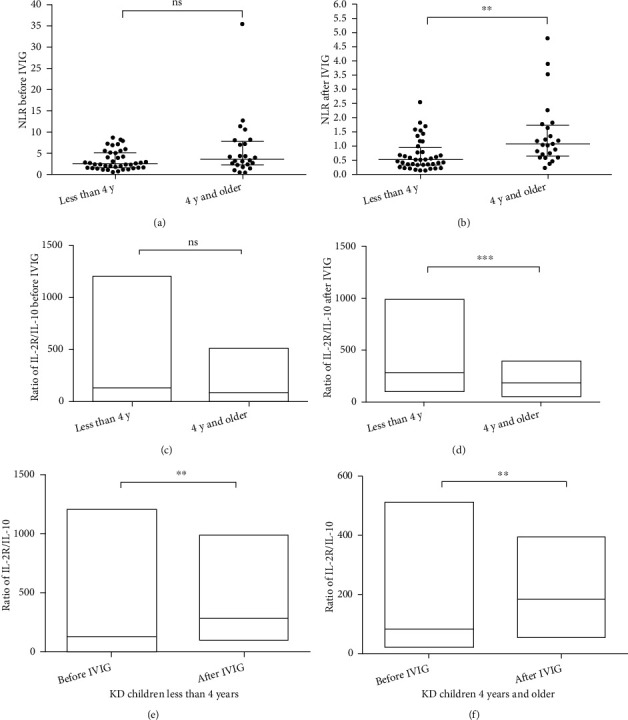
Effect of IVIG on the inflammatory response in children with KD under different conditions. (a) There was no significant difference in the NLR before IVIG between the group of patients with KD who were <4 years old and the group of patients who were ≥4 years old. (b) The NLR after IVIG in children with KD who were ≥4 years old was markedly higher than that in children with KD who were <4 years old. ^∗∗^*P* < 0.01. (c) No significant difference in the ratio of IL-2R/IL-10 before IVIG was observed between children with KD who were ≥4 years old and children with KD who were <4 years old. (d) The ratio of IL-2R/IL-10 after IVIG was lower in children with KD who were ≥4 years old than in children with KD who were <4 years old. ^∗∗∗^*P* < 0.001. (e) In children with KD who were <4 years old, the ratio of IL-2R/IL-10 increased significantly after IVIG (317 ± 26.58) and was higher than that before IVIG (185.5 ± 32.45). ^∗∗^*P* < 0.01. (f) In children with KD who were ≥4 years old, the ratio of IL-2R/IL-10 increased after IVIG (193 ± 17.26) and was higher than that before IVIG (124.5 ± 22.6). ^∗∗^*P* < 0.01. CD: cluster of differentiation; IL: interleukin; IVIG: intravenous immunoglobulin; KD: Kawasaki disease; NLR: neutrophil and lymphocyte ratio; ns: not significant; y: years.

When comparing the two different age groups, higher levels of NLR were found in children aged ≥4 years old compared with children with KD who were <4 years old. Before IVIG, the median NLR in older children with KD (3.65; *n* = 24) was higher than that in younger children with KD (2.59; *n* = 40), although no significant differences were observed. After IVIG, both NLR of the two groups decreased. The NLR of the older children with KD decreased to 1.06 and that of the other group was 0.49. There was a significant difference in the levels of NLR between the two groups (*P* < 0.01; Figures 1(a) and 1(b)).

One of the major characteristics of KD during the acute phase is hypercytokinemia. However, to the best of our knowledge, the underlying etiology and pathophysiology of KD remain unclear [13, 19]. Cytokine data were collected. To precisely determine the inflammatory state, the ratio of IL-2R/IL-10 in children with KD was used. Subsequently, the present study examined the ratio in the two groups of patients with different ages before or after IVIG. As shown in Figure 1(c), prior to IVIG, the median of the ratio was 131.7 in the younger children with KD and 80.49 in the group of children with KD who were ≥4 years old (not significant; Mann-Whitney test). After IVIG, the median of the ratio of IL-2R/IL-10 was 284.2 in younger children with KD and 180.8 in older children with KD. A markedly lower value was observed in children with KD aged ≥4 years compared with that in the group of younger children with KD (Mann-Whitney test; *P* < 0.001; Figure 1(d)).

After IVIG, the ratios of IL-2R/IL-10 in the two groups of children with KD were markedly increased compared with those before treatment (Figures 1(e) and 1(f)). Among the children with KD who were <4 years old, the mean ± standard deviation of the ratio of IL-2R/IL-10 was 185.5 ± 32.45 before IVIG and 317.7 ± 26.58 after IVIG. There was an obvious increase in the ratio after the therapy (*P* < 0.01). Children with KD aged ≥4 years had a similar response. Before IVIG, the ratio of IL-2R/IL-10 was 124.5 ± 22.6, and the ratio increased significantly to 193 ± 17.26 after IVIG (*P* < 0.01; paired *t*-test).

**Figure 2:**
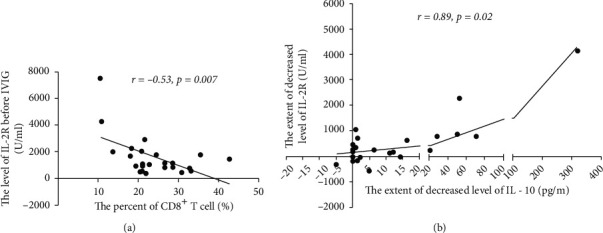
In children with KD who were ≥4 years old, there was a close correlation between the levels of IL-2R and the percentage of CD8^+^ T cells and the level of IL-10. (a) The levels of IL-2R before IVIG in children with KD who were ≥4 years old were negatively correlated with the percentage of CD8^+^ T cells. (b) The extent of the decrease of the levels of IL-2R was positively correlated with the extent of the decrease of the levels of IL-10.

In children with KD who were ≥4 years old, the levels of IL-2R before IVIG and the percentage of CD8^+^ T cells were analyzed, and a close negative correlation was identified (*r* = −0.53; *P* < 0.01; Figure 2(a)). As expected, in the group of children with KD, who were ≥4 years old, there was a close positive relationship between the extent of the decrease of IL-2R and IL-10 (*r* = 0.89; *P* < 0.05; Figure 2(b)).

### 3.3. Decreased Levels of IL-2R and IL-10 in Children with KD Are Associated with the Percentage of CD8^+^ T Cells and CD4^+^ T Cells during the Acute Phase When Treated with IVIG

**Figure 3:**
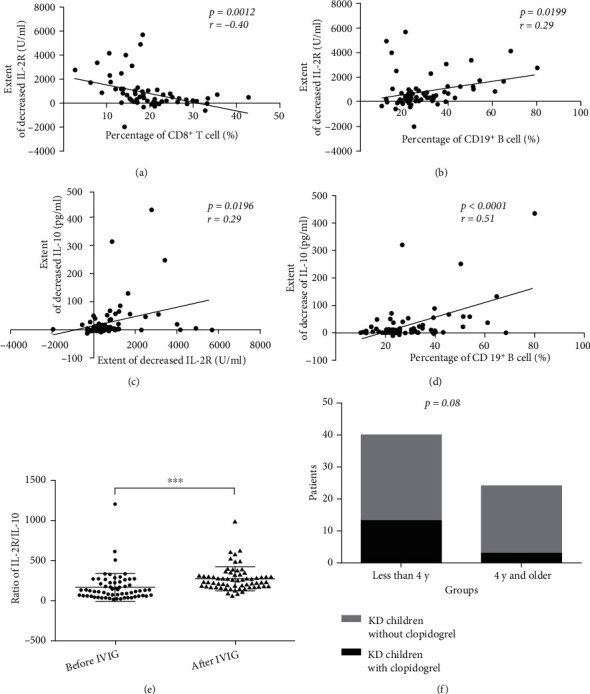
Association between altered levels of IL-2R and IL-10 and the percentages of CD8^+^ T and CD19^+^ B cells in all children with KD during KD and the distribution of KD children treated with clopidogrel between children with KD who were <4 years and children with KD who were ≥4 years. (a) Decreased levels of IL-2R were negatively correlated with the percentage of CD8^+^ T cells in children with KD. (b) Decreased levels of IL-2R were positively correlated with the percentage of CD19^+^ B cells in children with KD. (c) The extent of the decrease of IL-2R was positively correlated with that of IL-10. (d) The extent of the decrease of IL-10 was positively correlated with the percentage of CD19^+^ B cells in children with KD. (e) The mean value of the ratio of IL-2R/IL-10 before IVIG treatment was lower than that after IVIG (^∗∗∗^*P* < 0.001), which were, respectively, 162.6 ± 22.16 and 271 ± 19.29. (f) There was no difference in the number of children with KD treated with clopidogrel between patients who were <4 years old and patients who were ≥4 years old.

Based on the aforementioned results, the investigation was further expanded to a total of 64 children with KD. It was revealed that the extent of the decrease of IL-2R was negatively correlated with the percentage of CD8^+^ T cells in children with KD before IVIG (Figure 3(a)). The correlation coefficient was -0.40 and the *P* value was <0.01. Additionally, a positive correlation was identified between the extent of the decrease of IL-2R and the percentage of CD19^+^ B cells or the degree of IL-10 reduction, and both coefficients were 0.29 with *P* value <0.05 (Figures 3(b) and 3(c)). In addition, the extent of the decrease of IL-10 was positively correlated with the percentage of CD19^+^ B cells among all children with KD in the present study (Figure 3(d)). The mean value of the ratio of IL-2R/IL-10 of all patients prior to IVIG treatment was 162.6 ± 22.16, while the value after IVIG was 271 ± 19.29, which was markedly higher than that before IVIG (Figure 3(e)).

Clopidogrel has been recommended as a therapeutic regimen in combination with standard dose of IVIG and a low dose of aspirin for children with KD with obvious coronary artery abnormalities [1, 4, 5]. Then, the present study investigated whether there was a difference in the number of children with KD treated with clopidogrel between the groups of younger and older patients with KD. As shown in Figure 3(f), there was no significant difference in the number of children with KD treated with clopidogrel between the two age groups, although there were fewer children in the older group.

### 3.4. Dynamic PLT Count in Children with KD Treated with or without Clopidogrel

PLT is a strong risk factor of coronary artery abnormalities [20]. Those children with KD who received treatment with clopidogrel orally exhibited coronary artery luminal dilation of >3 mm, as determined by echocardiography, or the PLT count reached levels >600 × 10^9^/l, which according to the guidelines indicated an increased tendency of platelet activation [3, 21, 22]. Since the children with KD were investigated based on treatment with or without the use of clopidogrel, the present study was aimed at examining the dynamic changes of the PLT count during the acute stage of KD with the development of inflammation.

**Figure 4:**
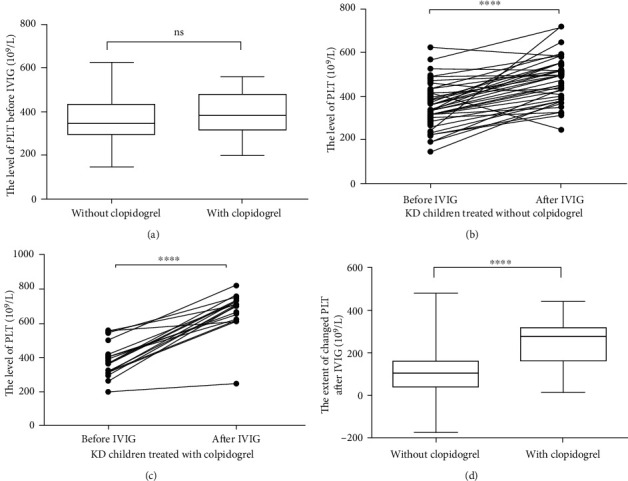
Altered PLT count in children with KD treated with or without clopidogrel. (a) Before IVIG, there was no obvious difference between the children with KD treated with and without clopidogrel. (b) In children with KD treated without clopidogrel, the PLT count markedly increased after IVIG. ^∗∗∗∗^*P* < 0.0001. (c) In children with KD treated with clopidogrel, the PLT count markedly increased after IVIG. ^∗∗∗∗^*P* < 0.0001. (d) After IVIG, the extent of the increase of the PLT count in children with KD treated with clopidogrel was markedly higher than that in children with KD treated without clopidogrel. ^∗∗∗∗^*P* < 0.0001.

There was no significant difference in the PLT count before IVIG between children with KD treated with and without clopidogrel (Figure 4(a)). Although all children with KD accepted the infusion of immunoglobulin in the acute stage of KD, there were still obvious increases in the PLT count after IVIG among most of the patients. In both children with KD treated with and without clopidogrel, significant increases in the PLT count were observed (*P* < 0.0001; Figures 4(b) and 4(c)).  Figure 4(c) unfolded the obvious rise in the PLT count during the period that followed the treatment of IVIG and just before the usage of clopidogrel. In Supplementary Table S6, patients' several times of the results of the PLT count after IVIG were listed to show the alteration of PLT. The count of PLT of all patients who received clopidogrel reached a level higher than 600 × 10^9^/l except just one child. The reason was that the exceptional child had occurred coronary artery abnormalities verified by echocardiography at the time the child was diagnosed as KD.

The patients treated with clopidogrel still had a markedly increased extent of PLT count after IVIG, which was 248.7 ± 31.38 (×10^9^/l; *n* = 16), while it was 106.1 ± 15.37 (×10^9^/l; *n* = 48) in patients who were not treated with clopidogrel (Figure 4(d)). When compared with each other, there was a significant difference between the two groups (*P* < 0.0001).

### 3.5. Levels of IL-2R and IL-10 and the Count of CD8^+^ T and CD19^+^ B Cells in Children with KD Treated with or without Clopidogrel

**Figure 5:**
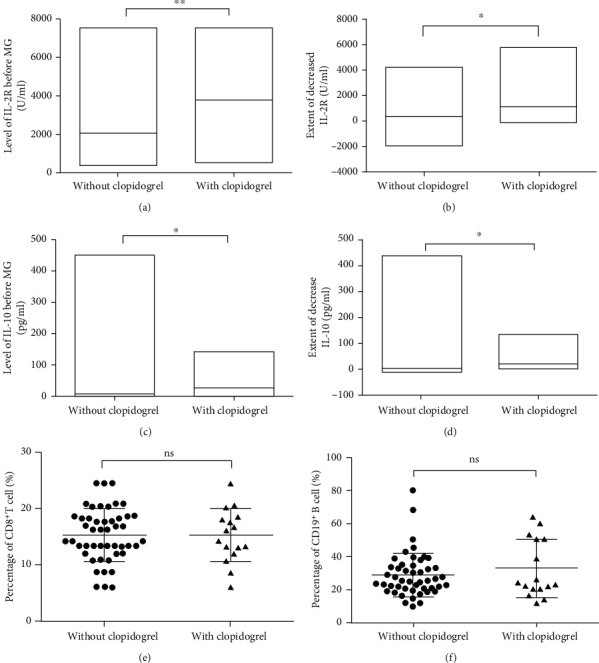
Levels of IL-2R and IL-10 in children with KD treated with or without clopidogrel. (a) The levels of IL-2R before IVIG in patients treated with clopidogrel were higher than those in patients who were treated without clopidogrel. ^∗∗^*P* < 0.01. (b) The extent of the decrease of IL-2R was significantly higher in children with KD treated with clopidogrel than that in those treated without clopidogrel. ^∗^*P* < 0.05. (c) The levels of IL-10 before IVIG in patients treated with clopidogrel were higher than those in patients who were treated without clopidogrel. ^∗^*P* < 0.05. (d) The extent of the decrease of IL-10 was significantly higher in children with KD treated with clopidogrel than that in those treated without clopidogrel. ^∗^*P* < 0.05. (e) There was no difference in the percentage of CD8^+^ T cells between the children with KD treated with and without clopidogrel, and the means and standard deviations were, respectively, 15.43 ± 0.68 and 15.43 ± 1.20. (f) There was no difference in the percentage of CD19^+^ B cells between the children with KD treated with and without clopidogrel, and the means and standard deviations were, respectively, 28.98 ± 1.92 and 32.96 ± 4.44.

An increase in PLT count in children with KD is usually associated with exacerbation of inflammation [23]. Therefore, the present study examined the altered levels of cytokines in patients treated with or without clopidogrel during IVIG therapy. The data demonstrated that children with KD treated with clopidogrel exhibited markedly higher levels of IL-2R before IVIG (3735 ± 595.4 U/ml) compared with patients treated without clopidogrel (2032 ± 254.3 U/ml; *P* < 0.01; Figure 5(a)). After IVIG, the extent of the decrease of IL-2R was still higher in children with KD treated with clopidogrel compared with that in children with KD treated without clopidogrel (Figure 5(b)). Similar results were also observed for the levels of IL-10. In children with KD treated with clopidogrel, higher levels of IL-10 before IVIG and a greater extent of the decrease of IL-10 after IVIG were observed compared with those in patients treated without clopidogrel (Figures 5(c) and 5(d)).

In children with KD, CD8^+^ T cells contribute to the inflammatory response through a cytotoxic mechanism, while B cells mainly function as antigen presenting cells [24–26]. The present study revealed a linear relationship between the percentage of CD8^+^ T cells and IL-2R and CD19^+^ B cells and IL-10. However, no differences were identified in the percentages of CD8^+^ T cells or CD19^+^ B cells between patients treated with and without clopidogrel (Figures 5(e) and 5(f)).

## 4. Discussion

KD is an acute febrile illness that involves systemic vasculitis of unknown cause, which is currently considered associated with an undetermined immune response to a common stimulus in genetically susceptible children. To the best of our knowledge, the underlying inflammatory mechanism has not been clearly elucidated [3, 13, 19]. The results of our study demonstrated that before and after IVIG, the extent of the decrease of the cytokine IL-2R was negatively correlated with the percentage of CD8^+^ T cells in children with KD and was positively correlated with the percentage of CD19^+^ B cells or the extent of the decrease of IL-10. No similar correlations were observed between lymphocyte subgroups and other cytokines, including IL-1, IL-6, TNF-*α*, or IL-1*β*. Therefore, the present following study focused on the relationship between IL-2R or IL-10 and lymphocyte subgroups.

Although the levels of IL-2R are not necessary for the activation of CD4^+^ T cells, their increase or decrease is indispensable for the mobilization of CD8^+^ T cells [27]. Exogenous IL-2 could lead to transient high levels of IL-2R, whose complex could form quickly and then drive naïve CD8^+^ T cells into active states [28]. In addition to IL-2 itself, IL-2R could be induced by various stimulators involved in the inflammatory response, including IL-1, IL-7, IL-12, TNF-*α*, and TNF-*β*, and could thus be stimulated to be rapidly and continuously expressed on the cell surface [29, 30]. Resting CD8^+^ T cells only express IL-2R*β* and *γ*-chains; however, when T cells are activated, the *α*-chain can be quickly induced to form a high affinity IL-2R [31]. Once this type of complete IL-2R is expressed on resting CD8^+^ T cells, proliferation and differentiation can be induced, leading the CD8^+^ T cells to develop into cytotoxic T cells. Additionally, IL-2R can be expressed on other types of T cell subgroups, such as T-helper 1 and CD4^+^ Treg cells [28, 32]. In patients under inflammatory conditions, markedly increased IL-2R levels lead to the release of IL-2R*α* in a soluble form from the cell surface to the peripheral blood [30, 33, 34], and the level of soluble IL-2R*α* was detected in our hospital. Therefore, the continually increasing levels of IL-2R in the peripheral blood of patients with KD suggest the presence of a progressive, enhanced T cell response, which sensitively reflects active cellular immunological inflammation.

However, the present results revealed that the percentage of CD8^+^ T cells before IVIG was clearly lower than the normal range. This may be attributed to the blood samples detecting T cell subgroups and the levels of cytokines being obtained at the same time in clinical practice, and thus, the difference was not detected in the T cell subgroups. When the levels of IL-2R increased in the acute phase in children with KD, the CD8^+^ T cells did not have enough time to be fully activated, and thus, the cells did not appear to proliferate. On the contrary, the evidence that the CD8^+^ T cell percentage was lower than the normal value exactly suggests the inhibition of the cellular response in acute inflammation in children with KD. Furthermore, the CD19^+^ B cell percentage was abnormally higher than the normal range, suggesting that B cell-mediated humoral immunity dominated the acute phase of KD. Based on these results, it could be concluded that the fluctuation of IL-2R was altered earlier than the activation of CD8^+^ T cells and is sensitive enough to indicate inflammation in children with KD.

IL-10 belongs to the family of anti-inflammatory cytokines and is mainly secreted by B cells [35]. IL-2R serves a critical role in promoting T cell proliferation and differentiation [36]. The present study revealed that after IVIG, the levels of both IL-2R and IL-10 were decreased compared with those before IVIG, suggesting that both cellular and humoral immunity were inhibited by IVIG. Considering the decreased degree of the above two cytokines was positively associated, the ratio of IL-2R and IL-10 was used to further evaluate the inflammatory status in children with KD to clarify the altered levels of IL-2R and IL-10. The data revealed that although there was an obvious decrease in the levels of IL-2R and IL-10, the ratio was increased after IVIG, indicating the more potent inhibitory effect mediated by IVIG on IL-10 and humoral immunity than IL-2R and cellular immunity.

Among these relationships, the positive correlation between IL-10 and the percentage of CD19^+^ B cells further verified our conclusion based on a previous study [12]. However, the previous data were limited to a group of children with KD who were ≥4 years old, which included fewer cases. The present study included all 64 children with KD, and the percentage of the lymphocyte subgroup, which was a relative number, was selected for analysis to exclude the difference among children with different ages. The results highlighted the role of IL-10 and IL-2R by demonstrating the number alteration and activity of CD19^+^ B cells and CD8^+^ T cells in the acute phase of KD, which reflected the degree of humoral or cellular immunity.

Furthermore, the levels of IL-2R and IL-10 fluctuate more flexibly and revealed more differences based on the variation of the microenvironment in children with KD compared with the PLT count or the percentages of CD8^+^ T cells and CD19^+^ T cells.  Figure 5 indicates the levels of both IL-2R and IL-10, revealing a marked difference between children with KD treated with clopidogrel and children not treated with clopidogrel under different types of conditions, including the phase when patients received IVIG before and the status after IVIG. While the PLT count of children with KD did not exhibit differences before IVIG. The continuous increase in the count after IVIG could not reflect the altered inflammatory response in children with KD, as did the percentages of CD8^+^ T and CD19^+^ B cells. In the acute phase of KD, the two subgroups of lymphocytes did not exhibit differences before IVIG between children treated with and without clopidogrel. Therefore, the PLT count and the percentages of CD8^+^ T and CD19^+^ B cells, unlike the levels of IL-2R or IL-10, are not sensitive enough to dynamically delineate the characteristics of the inflammatory response occurring in children with KD, nor able to predict the patients' response to IVIG. It was suggested that the detection and monitoring of IL-2R and IL-10 is more sensitive and convenient than that of the lymphocyte subgroups.

Regardless of the usage of clopidogrel, the PLT count of all children with KD increased continuously after IVIG, indicating the sustained activation of the platelets, which was consistent with previous reports [22, 23]. Some studies have reported that the number of platelets in the acute phase of KD tends to decrease [1, 37], which was not observed in the present 64 cases. Perhaps, there were no extremely critical cases in the present study. There is a phase during which platelets will start to increase in patients, and this lasts several weeks [21, 38]. This phenomenon may be attributed to the time of detection of PLT, which was after IVIG (72 h later), which is still in the active phase of platelets. IVIG neutralizes types of cytokines or other superantigens, but not platelets. The continuous rise of the PLT count is a proof of the active inflammatory cascading storm induced by altered levels of cytokines. Therefore, a delayed response in the dynamic PLT count may be a result of this phenomenon.

It suggested in one hand the necessity of clopidogrel. Clopidogrel exhibits its pharmacodynamics effect by specifically and irreversibly binding to P2Y12 and thus leads to the repression of platelet aggregation and the whole lifespan of the platelets [39]. Although additional therapy such as clopidogrel had been applied, the patients' activation of platelets enhanced continuously during the disease. If without clopidogrel, the degree of platelet's activation, the risk of coronary artery thrombosis, and thus induced inflammation would have risen more greatly.

On the other hand, Figure 4(d) showed that, even after IVIG, the extent of PLT in children with KD still increased. This phenomenon suggested the necessary treatment of starting the antiplatelet regimen including clopidogrel as early as possible. Among adults with coronary artery or cerebrovascular disease, the superior efficacy of the regimen combining aspirin with clopidogrel has been supported by several investigations compared with aspirin alone [3]. Despite the deficiency of the randomized clinical trials in children with KD, a lower incurrence of contiguous coronary artery lesions was observed after the combinative regimen performed than that of before using aspirin along. What is more, recommendations for clinical management of KD with coronary artery lesions published in 2020 in China advise that even the dilation of the arterial lumen recovers to normal or the coronary artery patency is reestablished, thrombosis, occlusion, and stenosis that occur in the coronary artery should be on the alert [5].

What is more, researches revealed that the anatomic variations are frequent in coronary artery branches and some luminal irregularities including abnormal decrease or dilation in luminal diameter could not become evident over several months. Therefore, the reported criteria of coronary artery abnormalities have been recognized to be arbitrary in some degree without considering the age or the size of patients [40]. And the study indicated that coronary dilation in KD children was more prevalent than previously reported [17, 41]. Therefore, patients who were found with coronary artery lesion by echocardiography should receive the therapy of clopidogrel as early as possible although in some cases the lesion was temporary. In addition, the patients with dilation of the coronary artery some time were not in a high level of PLT as shown in the Supplementary Table S6 even immediately after IVIG. However, as the natural dynamic course of PLT activation, the count of PLT would rise continuously in the following 3-4 weeks after IVIG. It suggested that a combinative consideration of the rising extent in the count of PLT with echocardiography should have a more powerful effect on the decision of clopidogrel usage than the way of judging by ultrasound.

The results demonstrated that there was no difference in the distribution of children with KD between the <4 and ≥4 years old age groups. Similarly, there was no difference found in the number of patients with KD between the clopidogrel and untreated groups. Therefore, it was suggested that there were no differences in platelet activation or occurrence of coronary artery abnormalities between children with KD who were <4 years old and who were ≥4 years old. Thus, an age of ≥4 years may not be a favorable factor for determining platelet activation and the occurrence of coronary artery abnormalities.

As for the NLR, which has been reported to have the ability to predict inflammatory conditions, such as IVIG resistance or development of coronary artery abnormalities in children with KD [21, 42], it reflected the intensity of stimulation and inflammation in children with KD, as well as the IL-2R/IL-10 ratio, particularly in patients aged ≥4 years. However, the ratio of IL-2R/IL-10 exhibited clear differences in patients before and after IVIG in both children with KD <4 and ≥4 years old, which verified its role as an index of the inflammatory response. When experiencing IVIG, the IL-2R/IL-10 ratio of all patients was higher than that before IVIG, revealing that T cell-mediated immunological inflammation became more dominant when considered in combination with the decreased NLR.

The results of the positive correlation between the decreased extent of IL-10 and IL-2R were obtained repeatedly. And the *P* values of the correlations in children with KD aged 4 years and older were higher than those in all 64 children with KD. The present investigation demonstrated that an age of ≥4 years was a beneficial factor for IVIG treatment of KD; however, children with KD who were 4 years and older did not benefit in terms of the occurrence of complications of coronary artery abnormalities or platelet activation, which was reported for the first time in the present study.

Despite the results were promising, there were still limitations within the present study. First, the data came from a retrospective study in a single center rather than a prospective one in multicenters, and the number of study population was not abundant enough to exclude biases. Second, there should have been more laboratory parameters to be evaluated before and after IVIG, such as the details of the coronary artery and monitoring of pharmacodynamics of clopidogrel. However, in some cases, frequent detections were not easy to be performed and obtained, including echocardiography and blood samples with consideration of the family's economic state and parents' degree of acceptance about the invaded detections. Therefore, the large size of the sample and the complete prospective design should contribute to estimate the precise inflammation state in children with KD. Based on patients' alterations in cytokines or coronary artery abnormalities, a predictive model should be an optimistic choice and worth exploring in our next project particularly designed for this issue.

## 5. Conclusions

The present retrospective study delineated the representative role of IL-2R combined with IL-10 in the development of KD course in different groups of children including age <4 years or ≥4 years and children treated with or without clopidogrel. At the same time, the PLT count, percentage of lymphocyte subgroups such as CD8^+^ T and CD19^+^ B cells, and NLR were also investigated. The results demonstrated the superiority of both the sensitivity and accuracy of IL-2R and IL-10 in indicating the degree of inflammation during KD. It further suggested a potentially necessary early usage of a therapeutic regimen involving antiplatelet agents, such as clopidogrel, as a protective treatment for coronary artery abnormalities, which are inclined to be damaged in KD.

## Figures and Tables

**Table 1:** Clinical and biochemical characteristics of the Kawasaki disease children.

Characteristics	Value	*P* value
Patients, *n*	64	—
Sex (male), *n* (%)	41 (64)	0.31^a^
Age, years	3.21 (1.25-4.75)	—
Body weight, kg	15.75 (11.00-18.11)	—
NLR before IVIG (<5.0)^b^	2.97 (1.74-5.65)	—
NLR after IVIG (<5.0)^b^	0.64 (0.36-1.19)	—
Percentage of CD19^+^ B cells before IVIG (NR, 6.8-15.8%)	25.46 (20.91-37.40)	<0.05
Percentage of CD8^+^ T cells before IVIG (NR, 18.2-32.8%)	15.43 ± 4.68	<0.01
Levels of IL-10 before IVIG, pg/ml (<9.1)	13.30 (6.02-38.88)	—
Levels of IL-10 after IVIG, pg/ml (<9.1)	4.9 (4.9-5.21)	—
Extent of decreased IL-10, pg/ml	6.80 (1.01-33.98)	—
Levels of IL-2R before IVIG, U/ml (223-710)	1705.50 (1109.00-2923.75)	<0.001
Levels of IL-2R after IVIG, U/ml (223-710)	1337.00 (881.25-1870.0)	<0.001
Extent of decreased IL-2R, U/ml	455.0 (130.75-1053.25)	—

^a^Compared with the data reported in the 2020JCS guide [1]. ^best^ according to the study published [14]. Data are presented as the mean ± standard deviation, number, or median (Q_L_-Q_U_). CD: cluster of differentiation; IL: interleukin; IVIG: intravenous immunoglobulin; NR: normal range; NLR: neutrophil lymphocyte ratio.

**Table 2:** Clinical and biochemical characteristics of children with Kawasaki disease in different age groups.

Characteristics	<4 years	≥4 years	*P* value
Patients, *n*	40	24	—
Sex (male), *n* (%)	27 (67.5)	14 (58.3)	0.46
Age, years	1.50 (0.85-2.81)	5.0 (4.25-6.00)	<0.01
Body weight, kg	11.65 (9.85-14.58)	18.45 (17.28-20.88)	<0.01
CD19^+^ B cells, %	28.86 (21.67-40.00)	22.30 (18.48-28.77)	<0.05
CD8^+^ T cells, %	16.82 ± 0.91	23.85 ± 1.57	<0.01
Extent of decreased IL-10, pg/ml	8.14 (1.47-39.93)	1.78 (0-15.80)	0.104
Extent of decreased IL-2R, U/ml	666.00 (229.25-1255.25)	243.5 (-35.5-757.75)	<0.05

CD: cluster of differentiation; IL: interleukin. Data are presented as the mean ± standard deviation, number, or median (Q_L_-Q_U_).

## Data Availability

The data that supports the findings of this study are available from Xinhua Hospital, but restrictions apply to the availability of these data, which are used under license for the current study, and so are not publicly available. Data is however available from the authors upon reasonable request and with permission of Xinhua Hospital.

## Supplementary Materials

Supplementary Materials Supplementary Table S1: the characteristics of patients with complete Kawasaki disease (KD) and incomplete KD. Supplementary Table S2: the main symptoms in patients with complete KD and incomplete KD. Supplementary Table S3: duration of fever till the retreatment of IVIG and the incidence of coronary artery abnormalities in children with KD. Supplementary Table S4: the individual characteristics of patients receiving retreatment of IVIG. Supplementary Table S5: the individual characteristics of patients receiving adjunctive therapies of corticosteroids. Supplementary Table S6: followed IVIG the alteration of patients' count of platelet (PLT) before usage of clopidogrel.
