# Supplementary material for: Predictive Role of IL-2R and IL-10 in the Anti-inflammatory Response and Antiplatelet Therapy of Kawasaki Disease: A Retrospective Study
Source: Mediators Inflamm. 2022 Feb 3;2022:4917550. doi: 10.1155/2022/4917550 (PMC8831045; doi:10.1155/2022/4917550)
Supplement: Supplementary Materials — Supplementary Table S1: the characteristics of patients with complete Kawasaki disease (KD) and incomplete KD. Supplementary Table S2: the main symptoms in patients with complete KD and incomplete KD. Supplementary Table S3: duration of fever till the retreatment of IVIG and the incidence of coronary artery abnormalities in children with KD. Supplementary Table S4: the individual characteristics of patients receiving retreatment of IVIG. Supplementary Table S5: the individual characteristics of patients receiving adjunctive therapies of corticosteroids. Supplementary Table S6: followed IVIG the alteration of patients' count of platelet (PLT) before usage of clopidogrel. [file 4917550.f1.docx]

**SUPPLEMENTARY INFORMATION**

**Predictive role of IL-2R and IL-10 in the anti-inflammatory response and anti-platelet therapy of Kawasaki disease: A retrospective study**

Zhang Chun^1^*^#^, Chen Lun^1^*, Chen Sun^2^, Bian Yan^2^, Shen Jia^2^, Zhang Peng^3^ Song JiaNi^1,4^

^1^ Department of Pharmacy, Xinhua Hospital affiliated to Shanghai Jiao Tong University School of Medicine, Shanghai 200092, China

^2^ Department of Pediatric cardiology, Xinhua Hospital affiliated to Shanghai Jiao Tong University School of Medicine, Shanghai 200092, China

^3^ Department of laboratory Medicine, Xinhua Hospital affiliated to Shanghai Jiao Tong University School of Medicine, Shanghai 200092, China

^4^ Department of Pharmacy, Shanghai University of Medicine &Health Sciences, Shanghai 201318, China

**Supplementary Table S1. The characteristics of patients with complete Kawasaki Disease (KD) and incomplete KD**

|  | Patients (n) (n %) | Gender (male, n) (n %) | Age (months) | Duration of fever to the diagnosis (days) | Duration of fever till receiving the first dose of IVIG (days) | Patients occurred with coronary artery abnormalities (n), (n %) |
| --- | --- | --- | --- | --- | --- | --- |
| Complete KD | 40 (62.5) | 25 (62.5) | 31 (13.50-55.50) | 6 (5-7) | 6 (4.63-7) | 3, (7.5) |
| Incomplete KD | 24 (37.5) | 16 (66.7) | 39 (17.50-59.50) | 5 (5-7) | 6 (4-7) | 1, (4.17) |
| *p* value | -- | 0.82 | 0.62 | 0.96 | 0.99 | 0.57 |

**Supplementary Table S2. The main symptoms in patients with complete KD and incomplete KD**

| Type of KD | Erythematous rash in trunk | |  | Extremity changes | |  | Cervical lymphadenothathy | |  | Oral changes | |  | Bilateral bulbar conjunctival injection | |
| --- | --- | --- | --- | --- | --- | --- | --- | --- | --- | --- | --- | --- | --- | --- |
|  | patients (n) | n% |  | patients (n) | n% |  | patients (n) | n% |  | patients (n) | n% |  | patients (n) | n% |
| Complete KD (n = 40) | 36 | 90 |  | 33 | 29.17 |  | 31 | 77.5 |  | 40 | 100 |  | 38 | 95 |
| Incomplete KD (n = 24) | 9 | 37.5 |  | 7 | 83 |  | 10 | 41.67 |  | 21 | 87.5 |  | 19 | 79.16 |
| *p* value | ＜ 0.001*** | |  | ＜ 0.001*** | |  | ＜ 0.01** | |  | 0.049 | |  | 0.093 | |

**Supplementary Table S3. Duration of fever till the retreatment of IVIG and the incidence of coronary artery abnormalities in children with KD**

| Type of KD | Number of Patients receiving a second dose of IVIG (n, n %) | Numbers of patients occurring coronary artery abnormalities before the second dose of IVIG (n, n %) | Duration of fever after the second dose (mean days) | Duration of patients′ symptom after the second dose (mean days) |  | Numbers of patients receiving a third dose of IVIG (n, n %) | Numbers of patients occurring coronary artery abnormalities before the third dose of IVIG | Duration of fever after the third dose (mean days) | Duration of patients′ symptom after the third dose (mean days) |
| --- | --- | --- | --- | --- | --- | --- | --- | --- | --- |
| Complete KD(n=40) | 3, (7.5) | 0, (0) | 2 | 3.33 |  | 1, (33) | 1, (33) | 0 | 4 |
| Incomplete KD(n=24) | 2, (8.3) | 0, (0) | 0 | 2 |  | 0, 0 | 0 | 0 | 0 |
| *p* value | ＞0.99 | -- | -- | 0.6 |  | ＞0.99 | -- | -- | -- |

**Supplementary Table S4. The individual characteristics of patients receiving retreatment of IVIG**

| Type of KD | | Patient number | Age (m) | Weight (kg) | Duration of fever till diagnosis | Duration of fever until the first dose of IVIG (days) | Duration of fever until the second dose (hours) | Duration of fever until the third dose (hours) | The incidence of coronary artery abnormalities | Whether adjunctively treated with corticosteroids |
| --- | --- | --- | --- | --- | --- | --- | --- | --- | --- | --- |
| Complete KD | | 1 | 6 | 8.7 | 4 | 6 | 47 | 0 | - | No |
|  |  | 2 | 51 | 18.5 | 7 | 7 | 72 | 0 | - | No |
|  |  | 3 | 130 | 32.5 | 6 | 7 | 84 | 17 | + | Yes |
| Incomplete KD | | 4 | 19 | 9.4 | 5 | 5 | 41 | 0 | - | No |
|  | 5 | | 62 | 18.7 | 5 | 6 | 43 | 0 | - | No |

**Supplementary Table S5. The individual characteristics of patients receiving adjunctive therapies of corticosteroids**

| Type of KD | Patients | Age  (m) | Weight  (kg) | The occurrence of coronary artery abnormalities | The interval days before adjunctive therapy of corticosteroids after IVIG | | The interval days before adjunctive therapy of corticosteroids after patients diagnosed as KD | The interval days before adjunctive therapy of corticosteroids after the coronary artery abnormalities diagnosed | Whether retreated with IVIG |
| --- | --- | --- | --- | --- | --- | --- | --- | --- | --- |
|  |  |  |  |  | The number of IVIG | days |  |  |  |
| Complete KD | 1 | 11 | 9.8 | Yes | The first dose | 2 | 3 | 0 | No |
|  | 2 | 13 | 11 | Yes | The first dose | 2 | 3 | 0 | No |
|  | 3 | 130 | 32.5 | Yes | The second dose | 5 | 9 | -3 | Yes |
| Incomplete KD | 4 | 20 | 11 | Yes | The first dose | 0 | 0 | 1 | No |

**Supplementary Table S6. Followed IVIG the alteration of patients’ count of platelet (PLT) before usage of clopidogrel**

| Patients treated with clopidogrel | The count of PLT after IVIG (×10^9^/L^3^) | | | The occurrence of coronary artery abnormalities |
| --- | --- | --- | --- | --- |
|  | The first test | The second test | The third test |  |
| 1 | 413 | 615 | -- | **+** |
| 2 | 349 | 506 | 652 | - |
| 3 | 660 | -- | -- | **+** |
| 4 | 526 | 554 | 716 | - |
| 5 | 343 | 617 | -- | - |
| 6 | 665 | 744 | -- | - |
| 7 | 541 | 621 | -- | - |
| 8 | 713 | -- | -- | - |
| 9 | 572 | 757 | --- | - |
| 10 | 614 | -- | -- | - |
| 11 | 517 | 663 | 696 | - |
| 12 | 807 | 798 | 820 | - |
| 13 | 646 | 702 | 615 | - |
| 14 | 706 | 722 | -- | - |
| 15 | 625 | 641 | 732 | - |
| 16 | 248 | 352 | 456 | **+** |
